# Tumor-Treating Fields Alter Nanomechanical Properties of Pancreatic Ductal Adenocarcinoma Cells Co-Cultured with Extracellular Matrix

**DOI:** 10.3390/jfb16050160

**Published:** 2025-05-03

**Authors:** Tanmay Kulkarni, Sreya Banik, Debabrata Mukhopadhyay, Hani Babiker, Santanu Bhattacharya

**Affiliations:** 1 Department of Biochemistry and Molecular Biology, Mayo Clinic College of Medicine and Science, 4500 San Pablo Road South, Jacksonville, FL 32224, USA; kulkarni.tanmay@mayo.edu (T.K.); banik.sreya@mayo.edu (S.B.); mukhopadhyay.debabrata@mayo.edu (D.M.); 2 Department of Physiology and Biomedical Engineering, Mayo Clinic College of Medicine and Science, 4500 San Pablo Road South, Jacksonville, FL 32224, USA; 3 Department of Medicine, Division of Hematology and Oncology, Mayo Clinic College of Medicine and Science, 4500 San Pablo Road South, Jacksonville, FL 32224, USA

**Keywords:** tumor-treating fields, pancreatic ductal adenocarcinoma, extracellular matrix, nanomechanical properties, tumor microenvironment

## Abstract

Tumor-Treating Fields (TTFields), a novel therapeutic avenue, is approved for therapy in Glioblastoma multiforme, malignant pleural mesothelioma, and metastatic non-small cell lung cancer (NSCLC). In pancreatic ductal adenocarcinoma (PDAC), several clinical trials are underway to improve outcomes, yet a significant knowledge gap prevails involving the cell-extracellular matrix (ECM) crosstalk. Herein, we hypothesized that treatment with TTFields influence this crosstalk, which is reflected by the dynamic alteration in nanomechanical properties (NMPs) of cells and the ECM in a co-culture system. We employed an ECM gel comprising collagen, fibronectin, and laminin mixed in 100:1:1 stoichiometry to co-culture of Panc1 and AsPC1 individually. This ECM mixture mimics the in vivo tumor microenvironment closely when compared to the individual ECM components studied before. A comprehensive frequency-dependent study revealed the optimal TTFields frequency to be 150 kHz. We also observed that irrespective of the ECM’s presence, TTFields increase cell membrane stiffness and decrease deformation several-folds in both Panc1 and AsPC1 cells at both 48 h and 72 h. Although adhesion for AsPC1 decreased at 48 h, at 72 h it was observed to increase irrespective of ECM’s presence. Moreover, it significantly alters the NMPs of ECM gels when co-cultured with PDAC cell lines. However, AsPC1 cells were observed to be more detrimental to these changes. Lastly, we attribute the stiffness changes in Panc1 cells to the membrane F-actin reorganization in the presence of TTFields. This study paves a path to study complex PDAC TME as well as the effect of various chemotherapeutic agents on such TME with TTFields in the future.

## 1. Introduction

Tumor-Treating Fields (TTFields) have gained significant attention in the last decade in the fight against cancer [1,2,3]. According to the National Cancer Institute, in 2022, cancer has become the second leading cause of death worldwide [4]. Increasing cancer incidence, therapeutic complications from the current standard-of-care treatments, and developing resistance to therapy [5] have led to the development of novel therapies such as the TTFields. This technology utilizes alternating electric fields at specific frequencies to the cancer-affected part of the body. Prior studies have shown that TTFields target tumor sites and inhibit mitosis and hence, the proliferation of tumor cells [6,7,8]. In addition, studies with TTFields have also demonstrated downregulation of DNA damage response [9,10], enhancement of antitumor activity [11], and interference with cell movement and migration [2]. This unique treatment modality stands out from the traditional therapies as it is non-invasive and tolerable [12] and has been approved by the Food and Drug Administration for the treatment of glioblastoma multiforme (GBM) and malignant pleural mesothelioma (MPM) [12], and most recently, for the previously treated metastatic small cell lung cancer (NSCLC) [NCT02973789]. Meanwhile, several clinical trials are either ongoing in patients with, gastric cancer (NCT04281576), hepatocellular carcinoma (NCT03606590) GBM (NCT02343549, NOVOTTF-200A, and NCT03869242), brain metastasis from NSCLC (NCT02831959), MPM (NCT02397928), or recently concluded such as pancreatic adenocarcinoma (PANOVA-3 and NCT03377491) and ovarian cancer (NCT03940196).

Pancreatic ductal adenocarcinoma (PDAC) is one of the most lethal cancers worldwide. With late presentation, the 5-year survival rate drops significantly for PDAC patients [13]. Currently, common treatment types include chemotherapy, targeted therapy, local therapy with radiation, and surgery [14]. Despite the advancement in technology and science, the disease remains elusive. PDAC is highly desmoplastic due to the dynamic and complex interactions between the cells and extracellular matrix (ECM) proteins in a tumor microenvironment (TME) [15]. Targeting desmoplasia has taken precedence owing to elevated intra-tumoral pressure and minimal drug delivery to the tumor site [16]. Several ongoing clinical trials focus on gaining insights into the PDAC ECM [17]. The biggest fallacy in studying the PDAC ECM is that studies often fail to consider the general impact of cell–ECM constituents and focus on individual components. In our previous work, we demonstrated that the cells and the ECM (collagen and fibronectin) when co-cultured significantly alter their nanomechanical attributes compared to the individual components [15]. Our study demonstrated a simplified TME and showed the complex and dynamic nature of cell–ECM interactions. However, the influence of TTFields on cell–ECM interactions is unknown and yet to be determined.

In this study, we hypothesize that the TTFields influences both PDAC cells and the ECM in a co-culture system and alters their individual characteristics and nanomechanical properties. To test our hypothesis, we synthesized ECM gel comprising collagen, laminin, and fibronectin mixed in a stoichiometric ratio of 100:1:1, respectively. Further, we studied crosstalk between ECM and PDAC cell lines such as Panc1 and AsPC1 in a co-culture system. The crosstalk was evaluated based on the dynamic alteration in nanomechanical signatures of the cells in the presence or absence of the ECM complex. These signatures are acquired using the atomic force microscopy (AFM) and are increasingly gaining attention in basic biology processes such as distinguishing cancer and normal cells, endocytosis, cell phase, and cell–ECM interactions by several research groups including ours [15,18,19,20,21]. Therefore, the influence of TTFields on simplified TME shown here will pave the way to delve into and gain deeper insights into more complex TME. Moreover, the nanomechanical alterations also give us insights into mechanical integrity TME under the influence of TTFields. The PANOVA2 trial demonstrated significant efficacy to the combination of gemcitabine alone, gemcitabine, and nab-Paclitaxel with TTFields compared to chemotherapy alone which led to the global randomized PANOVA 3 trial [22]. A press release indicated that PANOVA3 has met its primary endpoint. A more in-depth understanding of the mechanism of efficacy of TTFields in PDAC TME is paramount.

## 2. Experimental Methods

### 2.1. ECM Gel Preparation

ECM gel was prepared according to our previous work [15]. Type I Collagen (PureCol^®^ Solution, 3 mg/mL (bovine) #5005) and fibronectin (0.5 mg/mL; #5050) were purchased from Advanced BioMatrix (Carlsbad, CA, USA). Cultrex 3-D Culture Matrix Laminin I (available at 6 mg/mL; # 3446-005-01) was purchased from R&D Systems (Minneapolis, MN, USA). All three ECM proteins were brought to a final concentration of 1 mg/mL. Collagen, fibronectin, and laminin were mixed in the 100:1:1 stoichiometry. The resulting mixture was then employed to coat a 22 mm glass cover slip at 37 °C for 45 min to form a uniform coating of ECM. Post incubation, excess ECM solution was aspirated, and a fresh solution was incorporated on the glass slips and incubated again. This process was repeated 9 times to form a 9-layer ECM gel. Respective cells were cultured post 9 layers.

### 2.2. Cell Culture

Pancreatic ductal adenocarcinoma (PDAC) cell lines such as Panc1 and AsPC1 were purchased from the American Type Culture Collection (ATCC) (Manassas, VA, USA) and used without further validations. These cells were cultured at 10^5^ counts on the ECM-coated plates in Gibco Dulbecco’s Modified Eagle media (DMEM) (Waltham, MA, USA) and Roswell Park Memorial Institute 1640 (RPMI 1640), respectively, and supplemented with 10% Fetal Bovine Serum (FBS) and 1% Penicillin Streptomycin. They were then incubated at 37 °C in a humidified 5% CO_2_ atmosphere overnight. Following this, these cells were subjected to various treatments.

### 2.3. TTFields Treatment

To evaluate the dynamic alteration in the nanomechanical attributes of the PDAC cells under the influence of TTFields, we used the Inovitro system (Novocure, Haifa, Israel). This setup comprises a generator supplying alternating current electric fields to the base plates kept inside the incubator and maintained at 37 °C and 5% CO_2_. Each base plate comprises eight ceramic well slots into which the ceramic wells comprising the samples are cultured. We incorporated a pre-sterilized 22 mm glass cover slip to mono- and/or co-culture cells and ECM. Various sensors to monitor resistance, current, and temperature were attached to the walls of each ceramic well through which the experimental parameters were consistently maintained and viewed in an attached computer. In this study, we employed electric fields ranging from 50 kHz to 300 kHz to study the frequency-dependent response of TTFields on ECM. For the cell culture study with PDAC, we used TTFields with a frequency of 150 kHz as per the literature [23]. On the other hand, control experiments were performed on the same setup including the base plates. However, these plates were incubated in the absence of TTFields and maintained at 37 °C and 5% CO_2_.

### 2.4. AFM

AFM is a surface mapping technique and relies on the interaction between the tip attached to the cantilever and the sample surface. As the tip is brought closer to the surface, the cantilever deflects when the Van Der Waal’s forces overcome. The deflection in the cantilever is converted into the force values and displayed as a force-separation (F-S) curve. Herein, we utilized the nanoindentation technique, which limits this interaction between the tip and the sample to a few milliseconds. All the AFM experiments were performed using Dimension ICON Scanasyst and the sample temperature was maintained at 37 °C with a temperature-controlled hot plate. Furthermore, we used a 5 μm bead attached to a cantilever with a manufacturer-suggested spring constant of 0.1 N/m. This tip was then calibrated on an empty glass slide in the presence of de-ionized (DI) water to overcome the hydrodynamic drag during the tip–sample interaction. The calibrated spring constant was observed to be 0.09 N/m and the deflection sensitivity at 1 kHz tapping frequency was observed to be 40.3 nm/V. At least 48 datapoints were acquired on three separate occasions cumulating into 144 total datapoints to calculate the nanomechanical attributes of various ECM compositions. Similarly, at least 12 cells were probed, each with 9 datapoints, over the nuclear membrane region to accumulate to 108 datapoints. The ramping parameters during the nanoindentation experiment were restricted to a ramp size of 5 μm, a ramp rate of 1 kHz, 256 samples/ramp, and an applied force of 5 nN according to our previous literature [15].

### 2.5. Confocal Microscopy

We employed an LSM 800 confocal microscope (Plan-Apochromat 63× oil objective; Carl Zeiss, Inc.) (Thornwood, NY, USA) to image dynamic changes in actin fibers in cell membrane regions in the presence and absence of TTFields treatment. Foremost, a 22 mm glass cover slip containing live cells was gently rinsed with PBS (pH 7.4) and fixed with 4% paraformaldehyde for 5 min at room temperature. They were then rinsed twice with PBS (pH 7.4). Following this, the samples were incubated in a dark environment for 30 min at room temperature while stained with phalloidin-iFluor 488 reagent (Cat #ab176753; purchased from abcam (Boston, MA, USA)) and diluted at 1:1000. The samples were then washed three times and mounted on a glass cover slide with Vectashield^®^ antifade mounting media with DAPI purchased from VectorLabs (Newark, CA, USA). These samples were let to dry overnight at room temperature followed by transferring them to 4 °C until imaged.

### 2.6. Data Analysis

We analyzed the F-S curves using Bruker’s Nanoscope v1.9 software. Each curve was foremost filtered to remove any noise followed by baseline correction. The curve was then fitted between 90 and 20% using the Derjaguin–Muller–Toropov contact mechanics model [24]. GraphPad Prism 10 was used to calculate statistical significance and plot figures.

## 3. Results

### 3.1. Application of TTFields to Study Cellular Nanomechanical Properties Using the AFM

**Figure 1:**
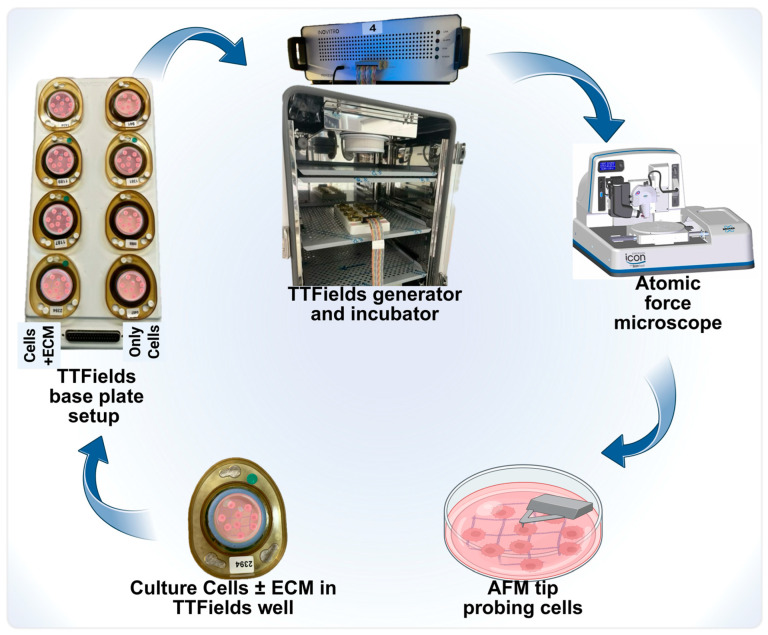
An experimental schematic of TTFields application to study AFM’s nanomechanical properties. Each of these wells was sealed with paraffin to prohibit the evaporation of the media due to the generation of heat. Further, customized experimental parameters were provided to each well in a base plate using a generator connected to the base plate. Post-TTFields treatment, these glass cover disks were gently removed and scanned under the AFM microscope to determine their nanomechanical change modulations.

TTFields utilize low-intensity electric fields to selectively target and inhibit tumor cell growth using a unique setup shown in Figure 1. This therapeutic regime has been shown to be safe and non-invasive [25]. Custom-built TTFields wells interfaced with various electrodes were used to culture cells. The cells were cultured on a 22 mm glass cover disk placed inside these wells. Eight such wells can be accommodated in one base plate as shown in Figure 1.

### 3.2. TTFields Alter Pancreatic Cancer Cell’s Nanomechanical Properties

**Figure 2:**
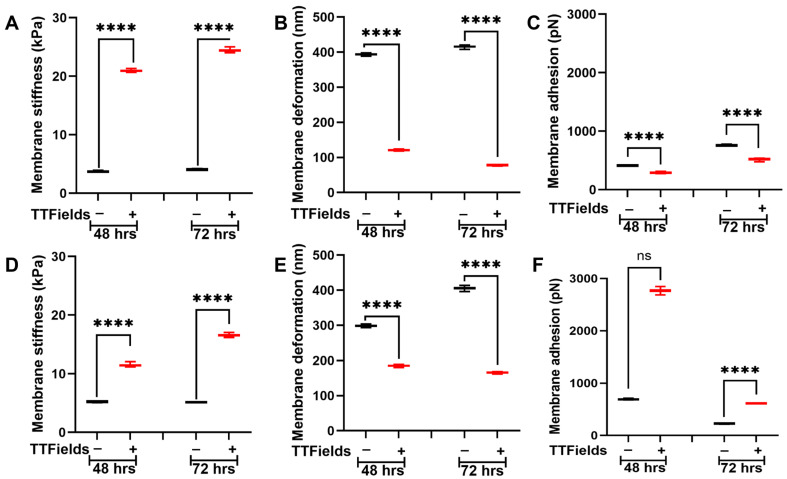
TTFields induce dynamic alteration in membrane stiffness in PDAC cell lines. The TTFields were operated at 150 kHz frequency. Panc1 cell membrane (**A**) stiffness, (**B**) deformation, and (**C**) adhesion. AsPC1 cell membrane (**D**) stiffness, (**E**) deformation, and (**F**) adhesion. (n = 12 cells; 144 datapoints). Statistical significance was performed using ONE-WAY ANOVA: ****, *p* < 0.0001.

AFM tip probes into the cell membrane, causing temporary indentation following the elasticity criteria. To acquire nanomechanical properties such as membrane stiffness, deformation, and adhesion, we utilized a 5 μm radius spherical tip designed to probe soft samples such as cells. We selected two different PDAC cell lines, namely Panc1 and AsPC1, primary and metastatic, respectively. We probed the membrane over the nuclear region. To measure the dynamicity, we opted for 48 h and 72 h. In the Panc1 cell line, we observed that the untreated cells exhibited an average membrane stiffness of 3.7 ± 0.1 kPa and 4.1 ± 0.1 kPa at the end of 48 h and 72 h, respectively, showing no significant change in their membrane stiffness as seen in Figure 2A. However, in the presence of the applied TTFields, we observed the membrane stiffness to elevate to 21 ± 0.2 kPa and 24.4 ± 0.3 kPa after 48 h and 72 h, respectively, as seen in Figure 2A. These results indicate that prolonged exposure of TTFields further increased the membrane stiffness. Deformation for untreated Panc1 cells after 48 h and 72 h was observed to be 393.6 ± 2.6 nm and 415.4 ±3.8 nm, respectively, as seen in Figure 2B. Care was taken to maintain deformation to less than 10% of the peak cellular height to avoid causing plastic deformations. In the presence of TTFields, we observed the deformation to decrease to 120.9 ± 1.9 nm and 77.7 ± 1.3 nm at the end of 48 h and 72 h, respectively, as seen in Figure 2B. These results indicate that stiffer cells become more resistant to deformation. Adhesion is another nanomechanical property commonly explored using the AFM. Adhesion indicates the repulsive pull the tip experiences as it proceeds to retract from the cell membrane surface. We observed that the untreated Panc1 cells exhibited an adhesion of 410.8 ± 5 pN and 758 ± 8.2 pN at the end of 48 h and 72 h, respectively. Upon the TTFields application, we observed that the Panc1 cell’s adhesion decreased significantly to 288.2 ± 10.2 pN and 519.4 ± 16 pN at 48 h and 72 h, respectively, as seen in Figure 2C.

The AsPC1 cells were subjected to TTFields followed by the AFM studies and the experimental parameters were kept consistent with that during the Panc1. We observed that the untreated AsPC1 cells displayed an average membrane stiffness of 5.3 ± 0.1 kPa and 5.1 ± 0.1 kPa at 48 h and 72 h, respectively, as seen in Figure 2D. We observed the AsPC1 cells to be stiffer than their Panc1 counterparts. Upon the TTFields application, AsPC1 cells were observed to become stiffer and exhibit average cell membrane stiffness of 11.5 ± 0.3 kPa and 16.6 ± 0.3 kPa at 48 h and 72 h, respectively. The prolonged exposure of TTFields displayed an additional increase in stiffness as seen in Figure 2D. Deformations at the end of 48 h and 72 h for the untreated AsPC1 cells were observed to be 298.3 ± 3.3 nm and 406.6 ± 5.1 nm, respectively. With TTFields application, it was observed to decrease to 185 ± 2.8 nm and 165.7 ± 2 nm, at 48 h and 72 h, respectively, as seen in Figure 2E. At 48 h, the untreated AsPC1 cells displayed an adhesion of 692.5 ± 10.2 pN which was observed to increase several-fold to 2768 ± 46.5 pN. Finally, at 72 h, the untreated AsPC1 cells exhibited an average membrane adhesion of 226.7 ± 5.9 pN, which increased to 611.5 ± 4.4 pN as seen in Figure 2F. These results indicate that TTFields alter membrane dynamics reflected in their nanomechanical alterations.

### 3.3. TTFields Alter Pancreatic Cancer Cell’s Nanomechanical Properties When Co-Cultured with the ECM

**Figure 3:**
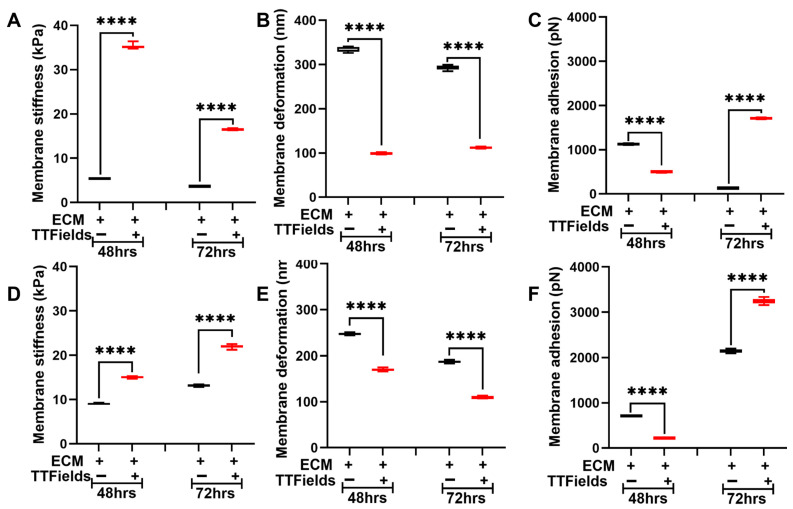
TTFields induce dynamic alteration in membrane stiffness in the PDAC cell lines in the presence of ECM. The TTFields were operated at 150 kHz frequency. Panc1 cell membrane (**A**) stiffness, (**B**) deformation, and (**C**) adhesion. AsPC1 cell membrane (**D**) stiffness, (**E**) deformation, and (**F**) adhesion. (n = 12 cells; 144 datapoints). Statistical significance was performed using ONE-WAY ANOVA: ****, *p* < 0.0001.

Cells and ECM mutually regulate each other’s nanomechanical properties and regulate TME homeostasis [26]. Herein, we co-cultured Panc1 and AsPC1 cells separately on an ECM comprising collagen, fibronectin, and laminin. For simplicity, we define these co-cultures as Panc1 + ECM or AsPC1 + ECM. We observed that after 48 h, the presence of ECM makes the untreated Panc1 cells stiffer compared to the absence of the ECM as seen in Figure 2A and Figure 3A. The average membrane stiffness of these cells was observed to be 5.4 ± 0.1 kPa. However, at 72 h, we observed that the untreated Panc1 + ECM made the cells softer (3.6 ± 0.1 kPa) compared to the Panc1 in the ECM’s absence as seen in Figure 2A and Figure 3A. This certainly proves that the cell–ECM interactions are complex and dynamic. Upon the application of TTFields, the average membrane stiffness of Panc1 + ECM at 48 h and 72 h was observed to be increased to 35.2 ± 0.4 kPa and 16.5 ± 0.1 kPa, respectively, as seen in Figure 3A. The deformation of Panc1 + ECM was observed to be 333.5 ± 5.1 nm and 292.5 ± 4.5 nm at 48 and 72 h, respectively. However, the TTFields n significantly decreased their deformation at 48 h and 72 h and was observed to be 99.3 ± 1.5 nm and 112.2 ± 1.6 nm, respectively, as seen in Figure 3B. Figure 3C shows the adhesion of the untreated Panc1 + ECM complex to be at 1128.2 ± 10.6 pN and 129.5 ± 3 pN at 48 h and 72 h, respectively, which were observed to exhibit contrasting trends in the presence of TTFields. At 48 h, we observed that the TTFields decreased the adhesion of the complex to 498.7 ± 9.5 pN compared to the absence of TTFields. However, at 72 h we observed a significant increase in Panc1 + ECM adhesion to be at 1707.9 ± 15.8 pN as seen in Figure 3C.

In the AsPC1 + ECM complex, the presence of the ECM significantly increased the cell membrane stiffness and was observed to be at 8.9 ± 0.2 kPa and 13.1 ± 0.2 kPa at 48 h and 72 h, respectively, as seen in Figure 2D and Figure 3D. Under the influence of TTFields, the average membrane stiffness of the AsPC1 + ECM complex significantly increased to 15 ± 0.2 kPa and 22 ± 0.4 kPa at 48 h and 72 h, respectively, as seen in Figure 3D. The deformation of the complex in the absence of TTFields was observed to be 247.3 ± 2 nm and 187.3 ± 2.5 nm at 48 h and 72 h, respectively. When the TTFields were applied, this parameter decreased consistently for 48 h and 72 h and was observed to be 169.6 ± 2.4 nm and 109.3 ± 1.6, respectively, as seen in Figure 3E. Adhesion exhibited contrasting trends at 48 h and 72 h. At 48 h, we observed that the application of TTFields decreased the adhesion from 711.4 ± 7.7 pN (untreated complex) to 221 ± 4.6 pN. On the contrary, 72 h of exposure with TTFields increased the adhesion from 2147 ± 38.6 pN (untreated complex) to 3248.3 ± 56.1 pN as seen in Figure 3F.

### 3.4. TTFields Alter ECM’s Nanomechanical Properties When in Co-Culture with Pancreatic Cancer Cells

**Figure 4:**
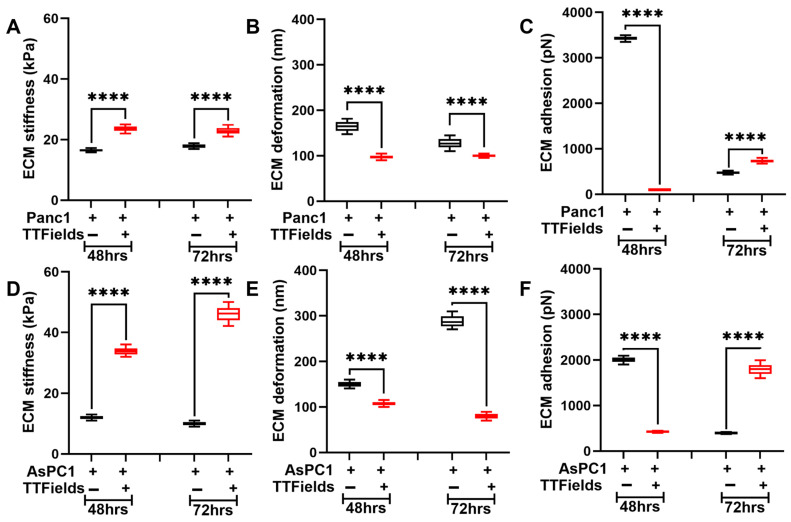
TTFields induce dynamic alteration in ECM stiffness in the presence of PDAC cell lines. The TTFields were operated at 150 kHz frequency. ECM in the presence of Panc1 cell: (**A**) stiffness, (**B**) deformation, and (**C**) adhesion. ECM in the presence of AsPC1 cell: (**D**) stiffness, (**E**) deformation, and (**F**) adhesion. (n = 144 datapoints). Statistical significance was performed using ONE-WAY ANOVA: ****, *p* < 0.0001.

Along with the pancreatic cancer cells, we monitored the dynamic alterations in the ECM complex’s nanomechanical properties in the presence and absence of pancreatic cancer cell lines and TTFields. Previously, we had shown that Panc1 and AsPC1 cells influence the nanomechanical properties of ECM and are dynamic in nature compared to ECM in the absence of these cells [15]. Foremost, we incorporated various combinations of ECM proteins originating from collagen, fibronectin, and laminin in the absence of any cell lines and measured their nanomechanical properties as seen in Figures S1–S3. We also measured the influence of various applied frequencies ranging from 50 to 300 kHz commonly employed in the TTFields paradigm. We observed significant alterations in collagen alone at various frequencies compared to the untreated. For almost all the applied frequencies, we observed a ~12-fold increase in average stiffness except for 150 kHz as seen in Figure S1A. For other combinations such as collagen + fibronectin (Figure S1B) and collagen + laminin (Figure S1C), we also observed dynamic alteration in their stiffness except for the collagen + fibronectin combination, wherein at 72 h, the change was insignificant for all the frequencies compared to the untreated. However, in the combination of collagen, fibronectin, and laminin at 150 kHz, we observed consistent and most decrease in the complex’s stiffness. This complex also yields a more appropriate TME environment compared to the other combinations. Both these reasons enabled us to further explore the complex’s nanomechanical properties in the presence of various PDAC cell lines. We also explored the deformation and adhesion parameters for various ECM complexes at varying TTFields frequencies and observed varying levels of changes in their properties as shown in Figures S2 and S3, respectively. After shortlisting the ECM complex stoichiometry and the TTFields treatment durations, we cultured PDAC cells separately on the ECM complex. The ECM stiffness in the presence of Panc1 cells and no treatment was observed to be 16.5 ± 0.4 kPa and 17.9 ± 0.6 kPa at 48 h and 72 h, respectively. Upon the TTFields application and in the presence of Panc1 cells, we observed this stiffness to increase at 48 h and 72 h and was at 23.5 ± 0.8 kPa and 22.8 ± 1 kPa, respectively, as seen in Figure 4A. This shows that with prolonged exposure of TTFields and in the presence of Panc1 cells, ECM shows no significant alteration in its stiffness. Deformation, on the other hand, for the ECM + Panc1 complex was observed to be 164.8 ± 10.3 nm and 127.8 ± 10.3 nm, respectively, in the absence of TTFields. Upon the application of TTFields, we observed a decrease in the deformation at both 48 h and 72 h and exhibited 97.1 ± 3.9 nm and 99.7 ± 3.1 nm, respectively, as seen in Figure 4B. Figure 4C shows adhesion observed to be 3428.8 ± 43.9 pN and 474.9 ± 26 pN at 48 h and 72 h, respectively. However, the application of TTFields at 48 h significantly decreased the ECM’s adhesion and was observed to be 98.9 ± 7 pN. On the contrary, at 72 h, the adhesion of the ECM + Panc1 complex was observed to be increased to 730.5 ± 38.1 pN as seen in Figure 4C.

Similarly, we evaluated ECM’s nanomechanical properties in the presence of the AsPC1 cells (ECM + AsPC1). Figure 4D displays the ECM stiffness in the absence of TTFields at 48 h and 72 h observed to be 12 ± 0.6 kPa and 10 ± 0.6 kPa, respectively. Under influence of TTFields, the stiffness of the ECM complex increased to 33.9 ± 1.1 kPa and 42.2 ± 2.2 kPa at 48 h and 72 h, respectively. The deformation of the ECM complex was observed to consistently decrease upon the application of TTFields as seen in Figure 4E. ECM complex exhibited a deformation of 150.3 ± 5.8 nm and 288.3 ± 12.1 nm at 48 h and 72 h, respectively. However, the TTFields reduced the deformation attribute to 107.3 ± 4.2 nm and 79.6 ± 5.7 nm at 48 h and 72 h, respectively. Figure 4F displays alteration in the adhesion of the ECM complex + AsPC1 cells. The application of TTFields at 48 h significantly decreased the adhesion from 2008.6 ± 55.6 pN to 426.5 ± 15.1 pN. At 72 h, TTFields significantly increased the adhesion of the ECM complex from 397.5 ± 13.2 pN to 1796.2 ± 112.4 pN as seen in Figure 4F. These results, coupled with Figure 3, strongly suggest the complex interactions between the cells and the ECM.

### 3.5. Confirmation of Stiffness Alterations in the Panc1 Cells Attributed to Dynamic Actin Rearrangement

AFM is a surface probing technique, suggesting that nanomechanical attributes such as stiffness arise from the cellular components in the membrane such as F-actin, microtubules, and intermediate fibers that are known to provide mechanical integrity to the cells and assist with proliferation and migration [27].

**Figure 5:**
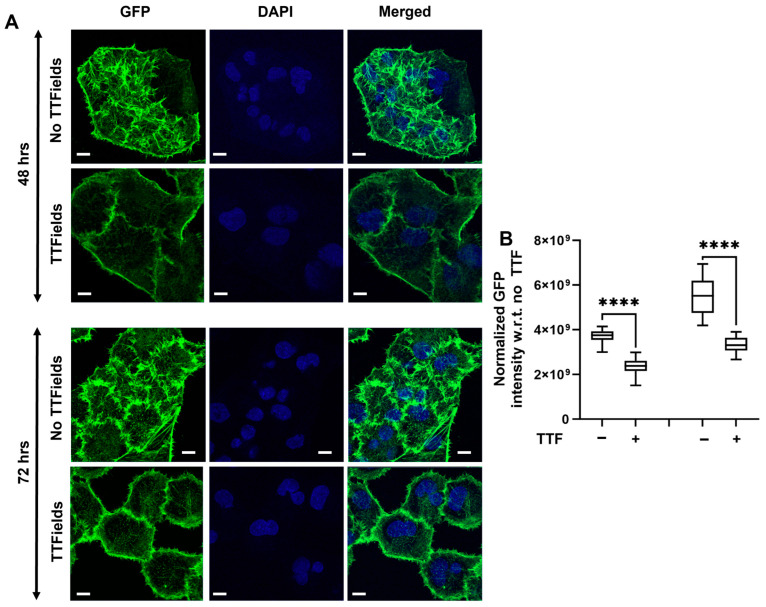
TTFields alter actin organization in Panc1 cells. The TTFields were operated at 150 kHz frequency: (**A**) Representative confocal images. (**B**) Quantification of GFP-labeled signal. (n = 12 cells) (Scale bar: 10 μm). Statistical significance was performed using ONE-WAY ANOVA: ****, *p* < 0.0001.

We monitored the morphology of the cells by focusing on the dynamic alteration in F-actin by staining it with phalloidin (green fluorescence signal) post cell-fixation. Under the confocal microscope, we observed that the Panc1 cells not subjected to the TTFields treatment displayed dense F-actin organization as evidenced by the long and prominent fibers seen in Figure 5A at both 48 h and 72 h. However, the TTFields were observed to disrupt the F-actin architecture and accumulate at the periphery of the cells as seen in Figure 5A. Moreover, the cells were observed to be enlarged including the nuclear region indicated by the DAPI stain. We further quantified the actin staining using a custom-built MATLAB program 2023a. Green fluorescence signal intensity signifying F-actin was found to be significantly decreased in the cells under the influence of TTFields as evident from the quantification (Figure 5B) in both 48 h and 72 h, further confirming our observations from the confocal images.

## 4. Discussion

TTFields, as the treatment modality, has been widely implemented in GBM cancer therapy [28,29,30,31]. However, in the pancreatic cancer paradigm, this modality is yet to be approved although ongoing clinical trials, and studies in preclinical models and in 2D culture are underway [13,22,32] and have shown promise. However, there is a significant knowledge gap in the use of TTFields in the PDAC paradigm. For instance, it is commonly known that the cells and the ECM in TME regulate each other’s properties and maintain homeostasis [33]. Although there are several studies understanding the biological and nanomechanical attributes of cells cultured on collagen or other ECM components, most of the studies fail to incorporate the various stoichiometry of ECM components to make it more accurate to the TME compared to a single ECM component [34,35]. Herein, we have used a combination of collagen, fibronectin, and laminin and two different PDAC cell lines such as Panc1 and AsPC1. To the best of our knowledge, this is the first study to incorporate such an ECM complex and gain insights into the nanomechanical properties resulting from the TTFields' influence on cell–ECM. It is well known that the cell–ECM interactions are dynamic in nature [36]. To incorporate the dynamicity, we considered two different time durations, namely 48 h and 72 h. Unlike collagen which is known to form fibers, fibronectin and laminin are known to form globular structures and often require collagen to adhere to [37]. Also, with changes in ECM stoichiometry, the complex's nanomechanical properties are known to alter [15]. Therefore, we foremost performed AFM on various combinations of the ECM to gather their nanomechanical properties to serve as a baseline as indicated in Figures S1–S3.

The ECM complex comprising collagen, fibronectin, and laminin under the influence of TTFields has never been studied before. Therefore, we performed an exhaustive study to gain insights into the nanomechanical alteration of various ECM complexes under a broad range of usable frequencies under the TTFields umbrella. It is crucial that we performed such extensive experiments that allowed us to shortlist the TTFields exposure frequency and time duration as well as the optimal ECM complex for further experiments involving the PDAC cells. Among all the ECM complexes, the one comprising collagen, fibronectin, and laminin resembles the closest with TME and pancreatic cancer stroma due to the availability of more ECM proteins. Furthermore, at 150 kHz for this complex, we observed consistent alteration in its stiffness over a period of 24–72 h. In addition, prior studies have been performed for 48 h and 72 h [38,39], so we decided to opt for these time durations. In Figure 2, Figure 3 and Figure 4, we consistently observed the stiffness and deformation trends to be inversely related. Predominantly, AFM is a surface mapping technique, and to retain the shape of the biological samples such as cells and fibers, we restricted the indentation to less than 10% of the cellular height [40] which has been widely accepted. As such, cellular membrane components such as actin, microtubules, and intermediate fibers come into play and govern the stiffness of the cell. Past studies have confirmed the role of these components in regulating membrane architecture, cellular integrity, and thus, stiffness [27,41,42]. Herein, through confocal microscopy, we confirmed that there was indeed a reorganization of F-actin in the cell membrane that caused alteration in the stiffness and made cells stiffer in the presence of the TTFields as shown in Figure 5. Deformation indicates how much the tip can indent into the sample. Since we are maintaining the experimental parameters such as applied force, ramp size, and ramp rate to be consistent during indentation, the stiffness of the cells provides resistance to the indentation, which is reflected in the deformation attribute. This explains the inverse relation between the stiffness and the deformation in a pure system such as 2D cell culture as seen in Figure 2, Figure 3 and Figure 4. Moreover, when we probe into the cell or the ECM, due to the transparency we can exactly probe on the region of interest. However, in the tissue sample, where the light from the objective does not pass through the sample, the tissue appears as a dark region. It will be a challenge to accurately know the landing region to be a cell or ECM. It will be interesting to see whether the inverse criterion holds true, especially when the probe lands on the cusp between the cell and the ECM.

Figure 4 shows the alteration in the ECM complex’s stiffness upon the application of TTFields. We consistently observed that irrespective of the cell lines, ECM stiffness increased several-fold under the influence of TTFields i. It remains to be seen what prompts these alterations exactly. However, the insights into ECM morphology would be interesting and exhaustive. We believe that the ECM stiffness increase could be due to the fiber rearrangement and change in the thickness and length of the fibers. Another possible explanation could be that the cells could be under stress due to the TTFields and pull the ECM, generating higher tensile stress. Nevertheless, it will be interesting to visualize the alterations in ECM complexes that will be performed in the future. Adhesion is seldom explored as it is often confused with the adhesion between the cell and the substrate. However, this adhesion, since it is measured at the membrane, comes from the repulsive pull experienced by the tip during its retraction. Past studies have attributed this property to cell adhesion molecules (CAMs) and integrins present at the cell membrane [43,44,45]. Herein, we observed that the adhesion parameter when the cells and ECM were co-cultured was lesser under the influence of TTFields compared to its absence at 48 h. Interestingly, the exact opposite trend in the adhesion was observed at 72 h as seen in Figure 3 and Figure 4. More work needs to be performed to deduce the exact mechanism behind these trends and possibly explore the CAMs and integrins.

Several research groups are conducting studies on the impact of TTFields in PDAC [1,46]. Most of the work focuses on the anti-mitotic effects of TTFields on PDAC cell lines [47,48]. The safety of TTFields has been demonstrated in a phase II study [49]. A combination of TTFields and radiation was found to be more effective than monotherapy in delaying PDAC progression [22]. Recently, impetus has been provided to the use of a combination of drugs combined with TTFields to optimize the therapeutic efficacy in PDAC [10]. These studies have shown promise; however, there is a plethora of knowledge yet to be fully understood in TTFields and PDAC such as resistance to the treatment, mechanisms of action behind TTFields as an effective therapeutic avenue, etc. To answer such questions, it is immensely vital to conduct experiments such as ours and understand how the TME is remodeled in the presence of TTFields. We acknowledge that the ECM complex we proposed is simplistic and far from the true TME; however, this work demonstrates a working model, and we are amidst optimizing the model by adding several other TME components stepwise to increase the complexity.

## 5. Conclusions

This work demonstrates the first of its kind on the influence of TTFields on the PDAC cell–ECM model and crosstalk. Alteration in the nanomechanical properties of the cells and the ECM in a co-culture system exhibits the dynamic and complex nature of the TME. TTFields alteration in cellular nanomechanical properties was attributed to the changes in actin reorganization. This study further paves the way to gain insights into the chemotherapy response in the presence of a cell–ECM co-culture system in the PDAC paradigm.

## Data Availability

The original contributions presented in the study are included in the article/supplementary material; further inquiries can be directed to the corresponding authors.

## Supplementary Materials

The following supporting information can be downloaded at https://www.mdpi.com/article/10.3390/jfb16050160/s1, Figure S1. Influence of various applied TTFields frequencies on different ECM compositions stiffness: (A) collagen; (B) collagen + fibronectin; (C) collagen + Laminin; (D) collagen + fibronectin + laminin. Collagen, fibronectin, and laminin were mixed in 100:1:1 stoichiometry. (n = 144 datapoints). Statistical significance was *p* < 0.0001 unless otherwise mentioned using # which corresponds to ns, *p* > 0.5. Figure S2. Influence of various applied TTFields frequencies on different ECM compositions deformation: (A) collagen; (B) collagen + fibronectin; (C) collagen + laminin; (D) collagen + fibronectin + laminin. Collagen, fibronectin, and laminin were mixed in 100:1:1 stoichiometry. (n = 144 datapoints). Statistical significance was *p* < 0.0001 unless otherwise mentioned using # which corresponds to ns, *p* > 0.5. Figure S3. Influence of various applied TTFields frequencies on different ECM compositions adhesion: (A) collagen; (B) collagen + fibronectin; (C) collagen + laminin; (D) collagen + fibronectin + laminin. Collagen, fibronectin, and laminin were mixed in 100:1:1 stoichiometry. (n = 144 datapoints). Statistical significance was, *p* < 0.0001 unless otherwise mentioned using # which corresponds to ns, *p* > 0.5.
