# Supplementary material for: Tumor-Treating Fields Alter Nanomechanical Properties of Pancreatic Ductal Adenocarcinoma Cells Co-Cultured with Extracellular Matrix
Source: J Funct Biomater. 2025 May 3;16(5):160. doi: 10.3390/jfb16050160 (PMC12112427; doi:10.3390/jfb16050160)
Supplement: Supplementary file 1 [file jfb-16-00160-s001.zip › jfb-3592751-supplementary.pdf]

Tumor-Treating Fields Alter Nanomechanical Properties of Pancreatic Ductal Adenocarcinoma Cells Co-cultured with Extracellular Matrix

Tanmay Kulkarni[1], Sreya Banik[1], Debabrata Mukhopadhyay[1,2] Hani Babiker[3*], and Santanu Bhattacharya[1,2**]

[1] Department of Biochemistry and Molecular Biology, Mayo Clinic College of Medicine and Science, 4500 San Pablo Road South, Jacksonville, FL, 32224, USA
[2]Department of Physiology and Biomedical Engineering, Mayo Clinic College of Medicine and Science, 4500 San Pablo Road South, Jacksonville, FL, 32224, USA
[3] Department of medicine, Division of Hematology and Oncology, Mayo Clinic College of Medicine and Science, 4500 San Pablo Road South, Jacksonville, FL, 32224, USA

Corresponding authors

*Hani Babiker
Department of medicine,
Division of Hematology and Oncology.
Mayo Clinic College of Medicine and Science,
4500 San Pablo Road South, Jacksonville, FL, 32224, USA

**Santanu Bhattacharya
Department of Biochemistry and Molecular Biology,
Mayo Clinic College of Medicine and Science,
Griffin 413, Mayo Clinic Florida,
4500 San Pablo Road South, Jacksonville, FL 32224, USA

Supplementary Figures

Figure S1.

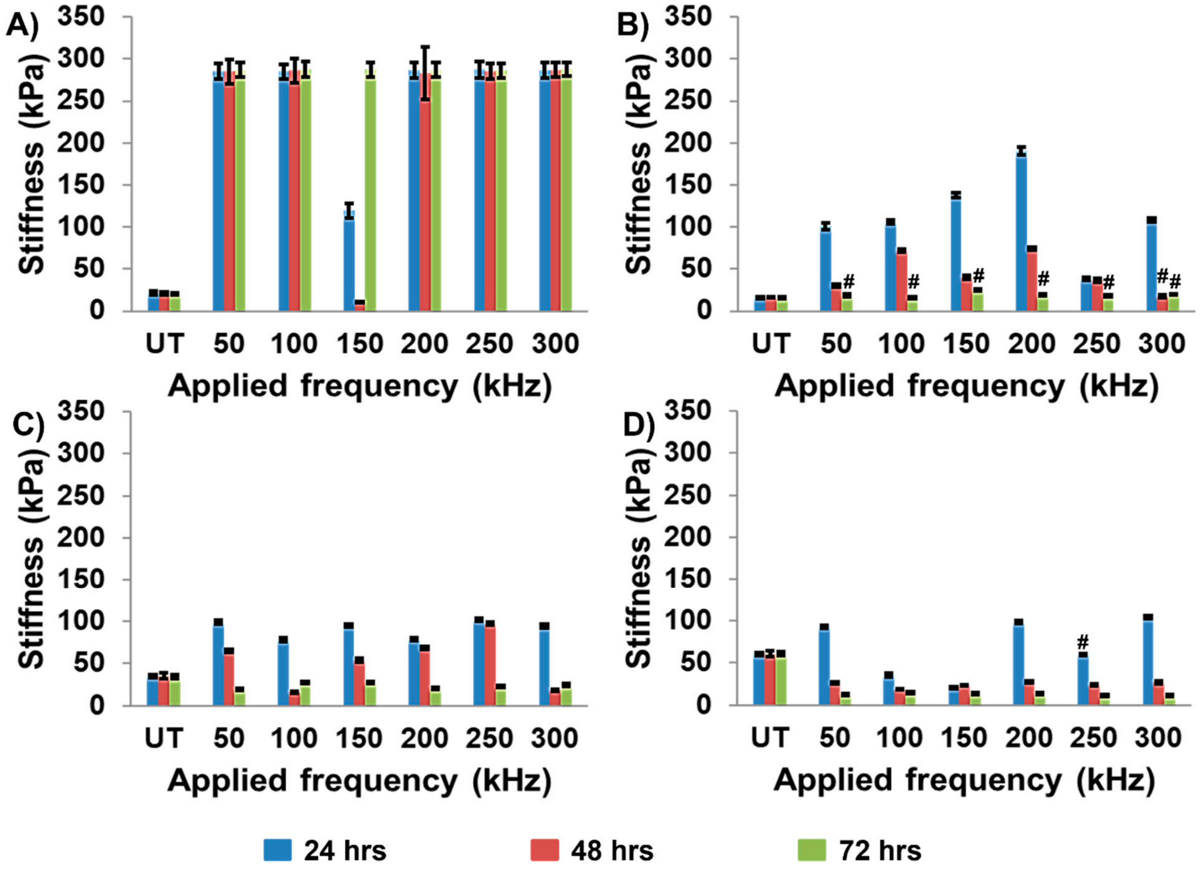

Figure S1. Influence of various applied TTFields frequencies on different ECM compositions stiffness. A) Collagen. B) Collagen + Fibronectin. C) Collagen + Laminin. D) Collagen + Fibronectin + Laminin. Collagen, Fibronectin and Laminin were mixed in 100:1:1 stoichiometry. (n=144 datapoints). Statistical significance was $p<0.0001$ unless otherwise mentioned using # which corresponds to ns, $p>0.5$.

Figure S2.

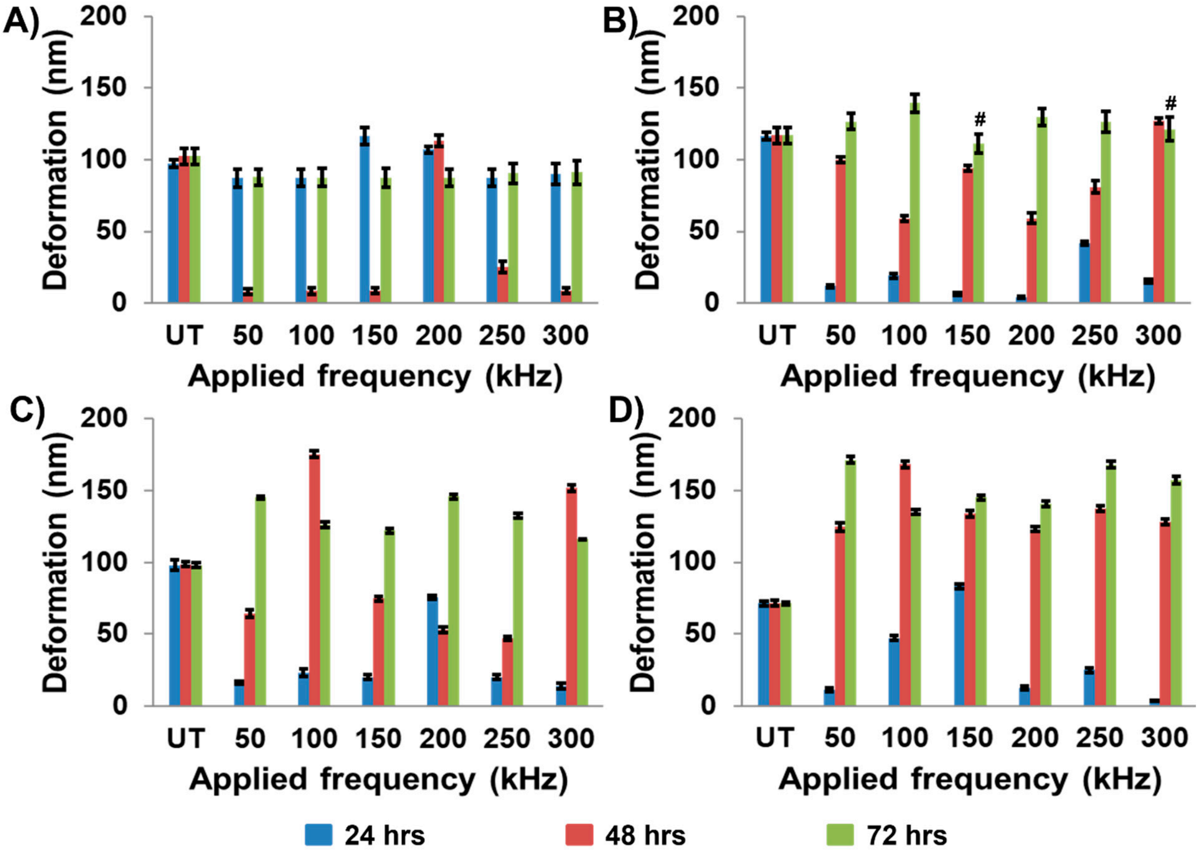

Figure S2. Influence of various applied TTFields frequencies on different ECM compositions deformation. A) Collagen. B) Collagen + Fibronectin. C) Collagen + Laminin. D) Collagen + Fibronectin + Laminin. Collagen, Fibronectin and Laminin were mixed in 100:1:1 stoichiometry. (n=144 datapoints). Statistical significance was $p<0.0001$ unless otherwise mentioned using # which corresponds to ns, $p>0.5$.

Figure S3.

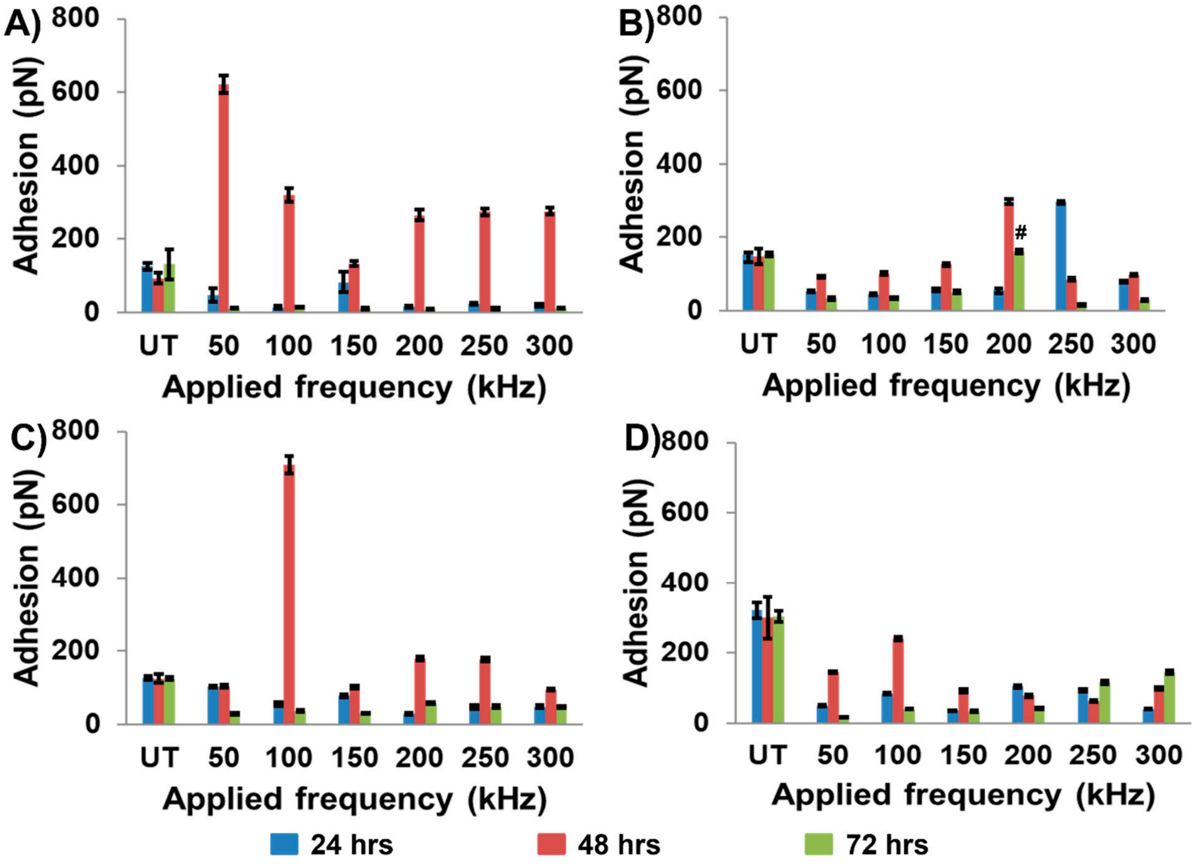

Figure S3. Influence of various applied TTFields frequencies on different ECM compositions adhesion. A) Collagen. B) Collagen + Fibronectin. C) Collagen + Laminin. D) Collagen + Fibronectin + Laminin. Collagen, Fibronectin and Laminin were mixed in 100:1:1 stoichiometry. (n=144 datapoints). Statistical significance was $p<0.0001$ unless otherwise mentioned using # which corresponds to ns, $p>0.5$.
